# Synthesis of Modified Magnetic Graphene Oxide with Mesoporous Silica for Extraction of the Pharmaceutical Compound Quercetin

**DOI:** 10.1155/2023/8581986

**Published:** 2023-09-19

**Authors:** Delnia Heidari, Soleiman Bahar

**Affiliations:** Department of Chemistry, University of Kurdistan, Sanandaj 66177-15175, Iran

## Abstract

In this study, magnetic mesoporous silica-Fe_3_O_4_-graphene oxide nanoparticles (Fe_3_O_4_@GO@mSiO_2_) were synthesized and used as sorbents for magnetic solid-phase extraction (MSPE) of trace amounts of quercetin in natural samples (spinach, green pepper, dill, and red onion). The sorbent produced was characterized by Fourier transform infrared (FTIR), scanning electron microscopy (SEM), energy dispersive X-ray spectroscopy (EDAX), X-ray diffraction (XRD), vibrating sample magnetometry (VSM), and X-ray photoelectron spectroscopy (XPS). The effects of various experimental factors on the percent recovery of quercetin, including extraction time, desorption time, sample solution pH, and adsorbent amount were investigated. The Fe_3_O_4_@GO@mSiO_2_ strategy showed excellent stability and sensitivity for the determination of quercetin, with a suitable linear range of 20–800 *µ*g L^−1^ and a detection limit of 5.2 *µ*g L^−1^. The data indicate that Fe_3_O_4_@GO@mSiO_2_ has a specific surface area and suitable adsorption capacity for the determination of quercetin.

## 1. Introduction

Quercetin is a flavonol found in many vegetables and fruits. In general, quercetin occurs in the form of aglycones and glycosides, mainly in leaves. Absorption of this compound occurs in the large and small intestine, where it undergoes glucuronidation, sulfidation, and methylation to improve its hydrophilicity. After metabolism, which takes place mainly in the intestine, it is distributed throughout the organism and excreted through feces, urine, and exhalation of carbon dioxide. Despite its cytotoxic effects in vitro, in vivo studies on animal models confirm its safety. Due to its anti-inflammatory properties and inhibition of radicals, this compound may protect against cancer, cardiovascular disease, chronic inflammation, oxidative stress, and neurological diseases [1]. However, to discover the potential of this flavanol, it is necessary to measure its bioavailability, which is significantly limited due to its hydrophobicity. In this context, nanoscience is one of the emerging research areas that has numerous applications in the fields of industry, science, ecology, engineering, chemistry, biomedicine, and pharmaceuticals [2, 3]. There are many traditional methods for extraction and adsorption of pharmaceutical compounds and active ingredients from plants. However, due to their complexity, high operating costs, and low efficiency, these methods have some limitations [4]. Recently, the use of nanotechnology as a promising tool has overcome these problems and limitations [5]. Nanomaterials have high adsorption capacity due to their large surface area and activity sites, so they have attracted much attention [6].

One of the most significant developments in nanotechnology is graphene and its derivatives, the so-called magnetic graphene oxide- (MGO-) based materials [7]. Graphene is a single-layer, two-dimensional (2D) nanomaterial in which the carbon atoms are honeycombed together by covalent bonds to form a flat sheet structure [3]. Unlike graphene, the exact structure of graphene oxide has not yet been determined, but several models have been proposed [8]. GO consists of various oxygen-containing functional groups, including hydroxyl, carboxyl, and epoxy groups [9], which are used as a suitable *Candida* adsorbent in the extraction and separation of organic and inorganic materials [10]. GO nanosheets adsorb insoluble molecules by noncovalent bonding via *π*-*π* interactions, but interfacial interaction with the matrix and insolubility are among their disadvantages [11]. It should be noted that the dispersion of GO nanosheets after adsorption is very high and their separation from solution is a very time-consuming and complicated process. To solve this problem, magnetic graphene nanocomposites are usually used [12]. The use of Fe_3_O_4_ nanoparticles on graphene results in a composite with magnetic properties that has numerous applications in biomedicine, magnetic fluids, drug delivery, nanomedicine, bioengineering, biotechnology, biosensors, cell imaging, energy storage and antifungal activity, catalysis, and material extraction [2, 11]. MGO has very favorable chemical and physical properties, including easy functionalization, high chemical and physical stability, oxidation specific surface area, size coordination, and surface binding active site [2].

Nowadays, magnetic Fe_3_O_4_ nanoparticles are widely used in the preparation of nanocomposite adsorbents, mainly due to their easy synthesis and numerous applications [6]. Meanwhile, pure Fe_3_O_4_ nanoparticles are easily oxidized in air and do not show high adsorption capacity in complex environments [13]. The vertical coating of mesoporous silica on graphene nanosheets can improve the surface properties of graphene [11].

In recent years, mesoporous silica nanoparticles (MSNs) have been developed and are increasingly used due to their properties such as mesoporous nature, easy functionalization, biocompatibility, nontoxicity, adjustable porosity, and stability [14]. The Stober method is the most widely used and suitable method to prepare micrometre-sized silica particles with spherical and monodisperse morphology, and this method pioneered the development of a chemical reaction for MSN [15]. These methods are known for acidic, basic, and neutral conditions, and by simply changing the reaction parameters, this synthesis approach leads to the formation of MSN with different shapes and sizes [16].

In the present work, an attempt was made to prepare an adsorbent of Fe_3_O_4_@GO@mSiO_2_ with graphene oxide properties as a nanosheet and porous mesoporous silica with magnetic properties by the Hammer method for the extraction of quercetin traces and subsequent spectrophotometry. These Fe_3_O_4_@GO@mSiO_2_ nanocomposites have large pores and voids that can significantly increase the surface area. The superparamagnetic properties of Fe_3_O_4_ contribute to the rapid separation of the adsorbent from the matrix solution. Based on these considerations, the preconcentration and determination of quercetin in the various real samples can be easily achieved.

## 2. Experimental Setup

### 2.1. Materials

Quercetin powder was supplied by Sigma-Aldrich Chemical Company (Missouri, United States). A stock solution of quercetin (100 *μ*g mL^−1^) in methanol was prepared and stored at 4°C before use. Methanol (MeOH), sulfuric acid, hydrochloric acid, sodium hydroxide, ethanol (EtOH), potassium permanganate (KMnO_4_), hydrogen peroxide (H_2_O_2_ 30%), ferric chloride hexahydrate (FeCl_3_.6H_2_O), ferric chloride hexahydrate (FeCl_2_.4H_2_O), cetyltrimethylammonium bromide (CTAB), ammonium nitrate, natural graphite powder, and tetraethyl orthosilicate (TEOS) were of analytical grade and purchased from Merck (Darmstadt, Germany). All reagents and solvents were used in this work as received without further purification.

### 2.2. Instrumentation

The ultraviolet-visible (UV-Vis) spectrophotometer, model SPECTROD 250 Analytic Jena from the USA, was used to record the absorption spectra. Fourier transform infrared (FTIR) analyzes were recorded using a Bruker Vector 22 spectrometer (Germany). SEM images and energy dispersive X-ray spectroscopy (EDAX) were performed using a field emission-scanning electron microscopy (FE-SEM) from Mighty-8 Instrument, TSCAN Company, Prague. The synthesized materials were also characterized by X-ray diffraction (XRD) and vibrational sample magnetometry (VSM).

### 2.3. Preparation of Graphene Oxide (GO)

GO was synthesized by the modified Hummer method. Briefly, 1 g of graphite and 23 mL of concentrated H_2_SO_4_ were added to a 250 mL conical flask with stirring in an ice bath. Then, 3 g of KMnO_4_ was added to the mixture. After a while, the ice bath was removed, and the resulting dark green solution was stirred at 35°C for one hour. Then, 46 mL of water was slowly added to the reaction mixture and placed in boiling water. The reaction mixture was kept at this temperature for another 30 minutes. Then, 10 mL of 30% H_2_O_2_ and 140 mL of distilled water were added to stop the reaction. The obtained precipitate was centrifuged and washed several times with 5% HCl and ethanol and dried in vacuo at 60°C.

### 2.4. Preparation of Fe_3_O_4_@GO Nanoparticles

The method for the synthesis of Fe_3_O_4_@GO nanoparticles was described by Wang et al. In summary, 0.1 g GO was kept in 150 ml deionized water containing 1.6 g FeCl_3_.6H_2_O and 0.6 g FeCl_2_.4H_2_O for 30 min at room temperature with stirring. Then, it was added dropwise to a 250 mL double-neck flask containing 25 mL of 30% ammonia solution with vigorous stirring. The solution was kept under nitrogen atmosphere at 25°C for 30 minutes and then heated to 80°C for one hour. The product was magnetically separated and washed with deionized water and ethanol, respectively. The Fe_3_O_4_@GO nanoparticles were dried in vacuo at 50°C.

### 2.5. Preparation of Fe_3_O_4_@GO@mSiO_2_

The synthesis of Fe_3_O_4_@GO@mSiO_2_ nanoparticles was described by Yin. Briefly, 50 mg Fe_3_O_4_@GO nanoparticles were ultrasonically dispersed in 50 mL deionized water containing 500 mg CTAB for 30 minutes. Then, 400 mL of deionized water and 50 mL of NaOH (0.01 M) were added and sonicated for 10 minutes. The mixture was heated at 60°C for 30 minutes. Then, 2.5 mL of TEOS/ethanol (v/v: 1/4) solution was added. The mixture was stirred at 60°C for 12 hours. The final product was magnetically separated and washed with deionized water. To remove CTAB, these nanoparticles were then mixed with 15 ml of methanol containing 120 mg of ammonium nitrate and stirred at 60°C for 12 hours. Finally, after 12 hours, they were separated with a magnet and washed several times with ethanol. The Fe_3_O_4_@GO@mSiO_2_ nanoparticles were dried in vacuo at 70°C.

### 2.6. Extraction Procedure

**Scheme 1:**
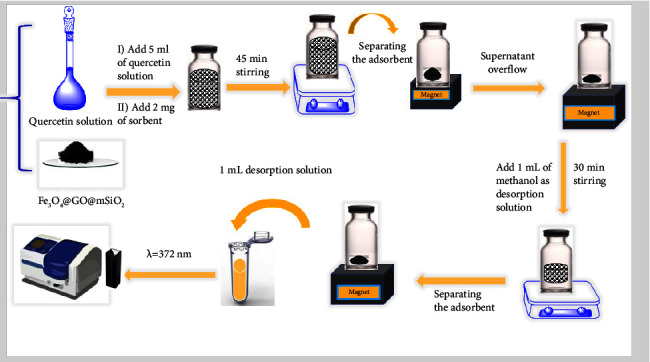
Schematic diagram of the proposed strategy for preconcentration of quercetin.

Adsorption was performed using 5 mL of quercetin sample solution at a concentration of 500 ng mL^−1^ (pH 8) and 2 mg Fe_3_O_4_@GO@mSiO_2_ as sorbent in a 10 mL glass vial for 45 minutes with magnetic stirring of 500 rpm at room temperature. Then, the solution was filtered with a magnet and washed several times with distilled water. Desorption was carried out with 1 mL of methanol to 2 mg Fe_3_O_4_@GO@mSiO_2_ for 30 min with a magnetic stirrer at 500 rpm. Finally, the solution was separated from the nanoparticles with a magnet and adsorption was measured with a spectrophotometer at 372 nm (Scheme 1).

### 2.7. Real Sample Preparation

The MSPE method was tested on real samples by analyzing samples of spinach, dill, green bell pepper, and red onion. These food samples were prepared for the solid-phase magnetic extraction method. All food samples were first washed with tap water and then with distilled water. Then, the samples were cut into cubes and exactly 5 g were weighed into 50 mL Falcon tubes. Then, 30 mL of methanol was added to each sample and stirred on a shaker for 24 hours. The samples were then centrifuged for 15 min, and the solid phase was separated from the liquids using an aqueous filter paper. The solvents were evaporated using a rotary apparatus, and the obtained extract was transferred to a 25 mL Falcon tube and diluted with distilled water after adjusting the pH [17].

## 3. Results and Discussion

The properties and characteristics of magnetic oxide-graphene nanoparticles coated with porous silica nanoparticles have been investigated using various techniques.

### 3.1. Characterization of Fe_3_O_4_@GO@mSiO_2_ Nanoparticles

**Figure 1:**
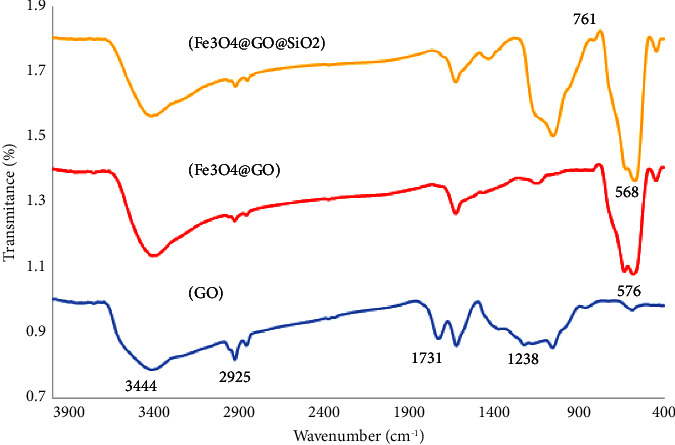
FTIR spectra of GO/Fe_3_O_4_@GO/Fe_3_O_4_@GO@mSiO_2_.

The chemical structure of Fe_3_O_4_@GO@mSiO_2_ was confirmed by FTIR spectroscopy, as shown in Figure 1. The FTIR spectra of Fe_3_O_4_@GO@mSiO_2_ showed the stretching vibrations of the O-H and C-OH groups on the graphene oxide sheets at 3444 cm^−1^ and 1238 cm^−1^, respectively. Also, the peak observed at 1731 cm^−1^ is related to the stretching vibration of the C=O bond, confirming the presence of carboxyl and carbonyl groups in the graphene oxide structure. The aliphatic C-H peak in the region of 2925 cm^−1^ is also visible in this spectrum. These peaks indicate the presence of epoxide, carboxyl, and hydroxyl groups in the structure of graphene oxide. Comparison of the spectra of graphene oxide and magnetic graphene shows a strong decrease in the peak of oxygen-containing functional groups in magnetic graphene compared to graphene oxide. The absorption peak at 576 cm^−1^ is related to the stretching vibration of Fe-O during the Fe_3_O_4_ preparation step. This peak confirms that Fe_3_O_4_ nanoparticles were successfully combined with graphene nanoparticles. Fe_3_O_4_@GO@mSiO_2_ showed three new peaks at 1047 cm^−1^, 761 cm^−1^, and 568 cm^−1^ compared to Fe_3_O_4_@GO, which relate to symmetric and asymmetric stretching vibrations and bending vibrations of Si-O-Si. The presence of these three peaks indicates the binding of mSiO_2_ to the surface of Fe_3_O_4_.

**Figure 2:**
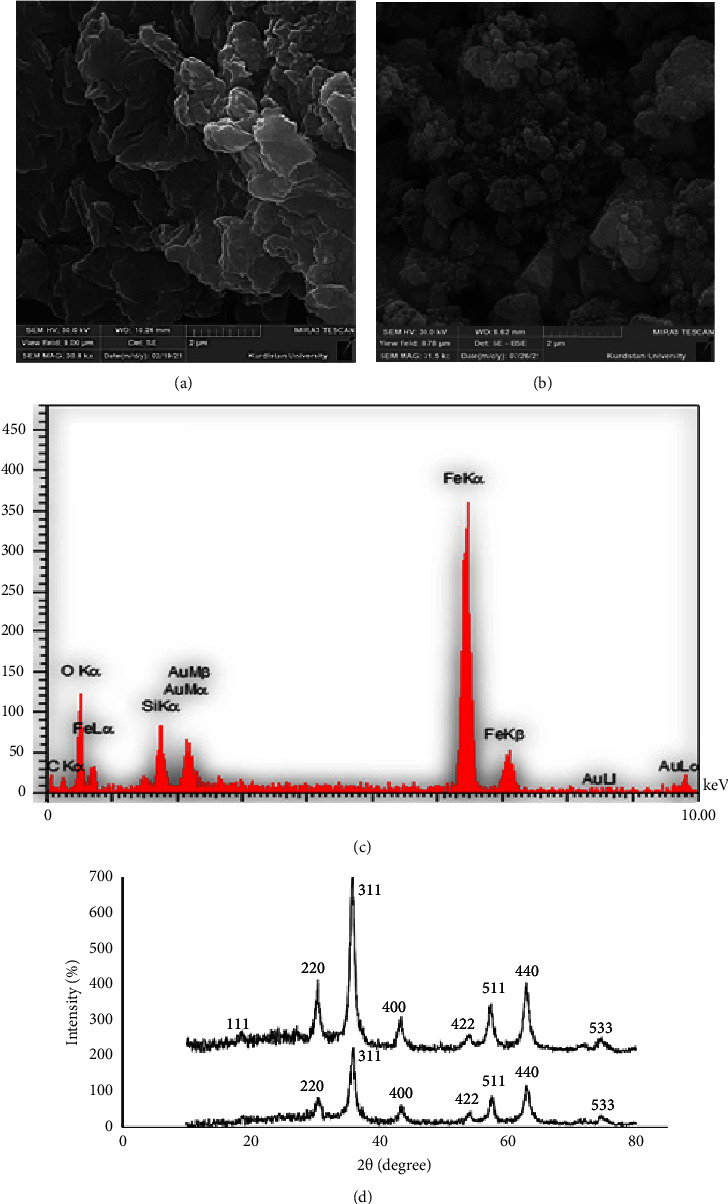
(a, b) SEM images of GO/Fe_3_O_4_@GO@mSiO_2_, (c) EDAX of Fe_3_O_4_@GO@mSiO_2_, and (d) XRD patterns for the Fe_3_O_4_@GO and Fe_3_O_4_@GO@mSiO_2_.

The surface morphology of GO, Fe_3_O_4_@GO@mSiO_2_ was studied by scanning electron microscopy (SEM). As shown in Figure 2(a), graphene oxide exhibits laminated sheets with many wrinkles, and after coating with Fe_3_O_4_ and mesoporous silica nanoparticles, the surfaces were completely roughened (Figure 2(b)).

The EDAX spectrum of Fe_3_O_4_@GO@mSiO_2_ was recorded to determine the elemental composition.  Figure 2(c) shows the energy dispersive X-ray spectroscopy (EDAX) spectrum of the sorbent, and the presence of the elements C, O, Fe, and Si can be found to be 33.8%, 8.26, 2.20%, and 81.21% (w/w), respectively. These results confirm the successful synthesis of Fe_3_O_4_@GO@mSiO_2_ as sorbent.

XRD analysis was used to evaluate the crystallinity of the prepared samples. In Fe_3_O_4_@GO, the diffraction peaks at 2*θ* angles are 52/30, 84/35, 46/43, 99/53, 58/57, 02/63, and 60/74 degrees, corresponding to crystal plates (220), (311), (400), (422), (511), (440), and (533), respectively. For Fe_3_O_4_@GO@mSiO_2_, the diffraction peaks at angles 2*θ* are about 52/18, 32/30, 71/35, 49/43, 93/53, 38/57, 94/62, and 54/74 degrees, and the crystal plates are (111), (220), (311), (400), (422), (511), (440), and (533). These peaks are consistent with the results reported in other research articles. No diffraction peaks of graphene oxide are seen in the diffraction patterns of Figure 2(d), and only specific peaks related to the crystal of magnetic iron oxide nanoparticles are visible. This may be due to the tiny amount of graphene oxide in the nanocomposite, or it may be that it peels and exfoliates between the graphene oxide layers during the synthesis process of crystal growth, and the graphene oxide diffraction peak disappears.

**Figure 3:**
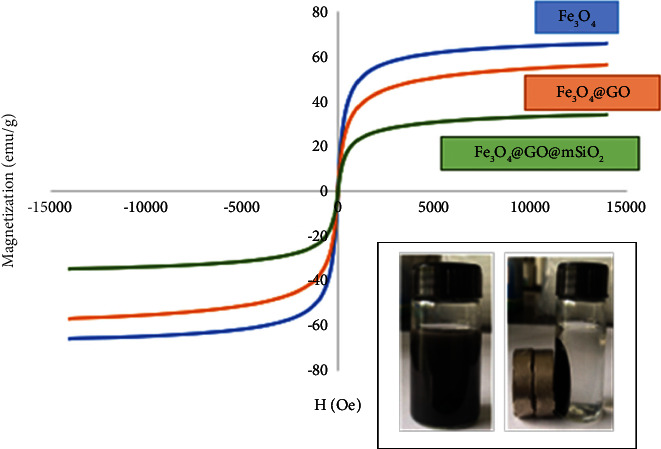
Magnetic hysteresis loops for Fe_3_O_4_/Fe_3_O_4_@GO/Fe_3_O_4_@GO@mSiO_2_.

The magnetic behavior of the Fe_3_O_4_@GO@mSiO_2_ nanosorbent was determined using a vibrating sample magnetometer.  Figure 3 shows the magnetic hysteresis loops of the samples, which are S-shaped and pass almost through the origin, indicating that the samples have near zero hysteresis magnetism. These results indicate the superparamagnetic behavior of the synthesized samples. The VSM measurements for Fe_3_O_4_ nanoparticles are (65/65 emu/g), Fe_3_O_4_@GO (emu 34/56), and Fe_3_O_4_@GO@mSiO_2_ (13/34 emu/g). Addition of graphene oxide and mesoporous silica to the nanoparticles reduces the saturation magnetization. The decreased saturation magnetization in nanocomposites may be related to the presence of nonmagnetic layers GO and mSiO_2_, the lower charge of Fe_3_O_4_ on the surface of mSiO_2_ and GO, the particle size of Fe_3_O_4_, and the electron exchange between Fe_3_O_4_ and GO and mSiO_2_.

**Figure 4:**
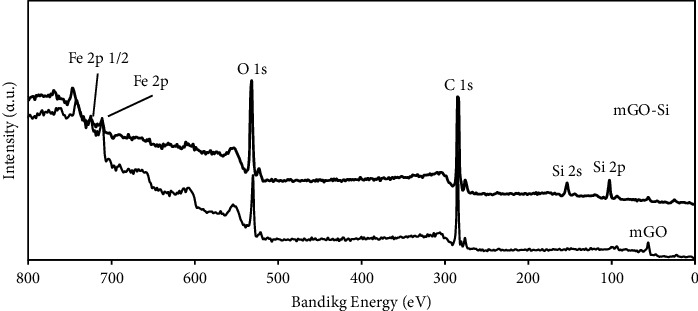
XPS spectra of mGO and mGO-Si.

The chemical status of the element in Fe_3_O_4_@GO and Fe_3_O_4_@GO@mSiO_2_ was further investigated by XPS (Figure 4). The positions of the C 1s, O 1s, and Fe 2p peaks can be seen in the spectrum of Fe_3_O_4_@GO. Compared with Fe_3_O_4_@GO, two additional peaks of Si 2s and Si 2p have also appeared in the full spectrum of Fe_3_O_4_@GO@mSiO_2_, which is a reason for the presence of mesoporous silica in the synthesized mesoporous silica magnetic graphene nanocomposite.

### 3.2. Optimization of MSPE Conditions

In order to optimize the magnetic solid-phase extraction method, various parameters such as extraction time, desorption time, adsorbent amount, and pH of the sample solution were investigated.

#### 3.2.1. Effect of Extraction Time and Desorption Time

**Figure 5:**
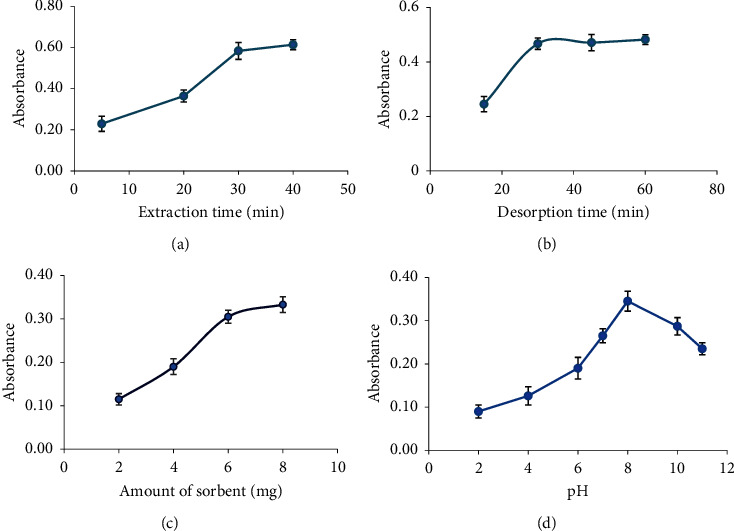
(a) Effect of adsorption time, (b) effect of desorption time, (c) effect of sorbent amount, and (d) effect of sample solution pH.

Optimization of extraction and desorption time is an essential and fundamental parameter affecting the sensitivity of extraction procedures. The extraction and desorption times were studied in a range from 5 to 40 min. The results in Figures 5(a) and 5(b) show that as the time increases, the efficiency of extraction and desorption of the analyte increases. As can be seen, the maximum extraction efficiency was reached after 40 min, and the maximum desorption time was 45 min. Finally, 40 min and 45 min were used as the optimum extraction and desorption times for further experiments.

#### 3.2.2. Effect of Sorbent Amount

The effect of sorbent amount on the preconcentration of quercetin was investigated in the range of 2.0–8.0 mg. As shown in Figure 5(c), the absorbance increased with increasing amount of sorbent, while the difference between the values of 6 and 8 mg of adsorbent was not very large. Therefore, based on the results, 8 mg of adsorbent can be selected as the optimum amount.

#### 3.2.3. Effect of Extraction Solvent Type

To evaluate the effects of the type of extraction solvent, methanol, ethanol, acetone, acetonitrile, and chloroform were used as solvents. The results showed that the highest amount of adsorption signals was associated with the use of methanol as solvent. The desired solvent must be able to dissolve the quercetin molecule and overcome the bond between quercetin and adsorbent. In addition, the extraction solvent must have the same polarity as the quercetin in order for the quercetin to be properly desorbed in the solvent. Due to the presence of hydroxyl groups, quercetin is considered a polar molecule, and considering the pKa value of quercetin (pKa: 6.9) at pH 8.0, the composition of quercetin is almost molecularly neutral. Based on the data obtained, methanol (aprotic solvent) was considered as the optimal extraction solvent for the desorption of quercetin and was used in the next experiments.

#### 3.2.4. Effect of Extraction Solvent Volume

The effect of the volume of the extraction solvent was also evaluated. The results showed that increasing the volume of extraction solvent causes a decrease in the amount of adsorbed signal, as the concentration of quercetin decreases with the increase in the volume of extraction solvent. These results showed that the volume of 1 ml of methanol is sufficient for the desorption of quercetin and that the highest concentration of the extracted sample can be obtained with this volume. It should be noted that although a higher concentration factor would be expected with smaller volumes of solvent, this was not used due to a significant error in the separation of quercetin from the sample solution. Finally, the volume of 1 mL of solvent was considered as the optimal extraction solvent volume and was used in the next experiments.

#### 3.2.5. Effect of Sample Solution pH

The effect of sample pH plays a significant role in SPE procedures. Therefore, the pH of aqueous sample solutions in the range of 2–11 was investigated. From the results (Figure 5(d)), the adsorption of quercetin gradually increased with increasing pH from 2 to 8 and then decreased at a higher pH. The possible adsorption mechanism between the sorbent and quercetin composition depends on the degree of protonation and deprotonation of quercetin and its interaction with the sorbent surface. Low adsorption in acidic environment may be due to the presence of abundant proton ions and protonation of quercetin hydroxyl groups with pKa = 6.9 and silanol groups on the sorbent surface and subsequent electrostatic repulsion between these groups. With a gradual increase in pH up to 8, the positive charge on the surface of the analyte and sorbent decreases, and hydrogen bonding may occur between them, ultimately leading to the highest amount of adsorption. In contrast, at a pH above 8, the adsorption decreases again, which may be due to the electrostatic repulsion in acidic environments between the negative charges of the sorbent surface and the deprotonated quercetin molecules. The highest adsorption was found at pH 8, where the strongest interaction between the sorbent and the quercetin composition occurs. Therefore, pH 8 was chosen as the optimum pH for further work.

#### 3.2.6. Extraction Capacity

**Figure 6:**
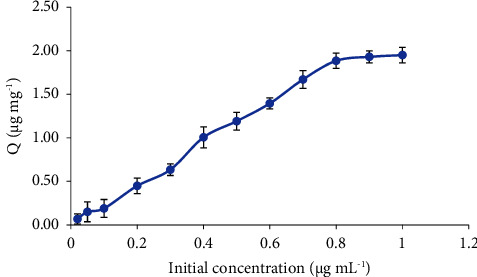
Extraction capacity curve of Fe_3_O_4_@GO@mSiO_2_ for quercetin.

The extraction capacity of the proposed Fe_3_O_4_@GO@mSiO_2_ nanosorbent was calculated using equation (1) and a series of aqueous quercetin solutions ranging from 1 to 0.02 *μ*g mL^−1^. As shown in Figure 6, the amount of extracted quercetin increases with increasing initial quercetin concentration up to 0.8 *μ*g mL^−1^ and then remains constant. The extraction capacity of Fe_3_O_4_@GO@mSiO_2_ nanoparticles was calculated to be 1.89 mg g^−1^.

(1) $$\begin{array}{c} Q=\frac{\left(C_{\text{ext}}\;V_{\text{analyte}}\right)}{m}\text{.} \end{array}$$

In equation (1), *Q* (mg g^−1^) is the adsorption capacity of Fe_3_O_4_@GO@mSiO_2_, C (*μ*g L^−1^) is the extracted concentration of quercetin obtained by the calibration line equation, *V* (mL) is the volume of quercetin solution, and *m* (mg) is the weight of the Fe_3_O_4_@GO@mSiO_2_.

### 3.3. Isotherm Study

The adsorption mechanism of quercetin in Fe_3_O_4_@GO@mSiO_2_ was evaluated by using two isotherm models, Langmuir (equation (1) and Freundlich (equation (2)).

(2) $$\begin{array}{c} \frac{C_{e}}{q_{e}}=\frac{1}{q_{\max}k_{L}}+\frac{C_{e}}{q_{\max}}\text{,} \end{array}$$

(3) $$\begin{array}{c} \log\;q_{e}=\log\;k_{F}+\frac{1}{n}\log\;C_{e}\text{,} \end{array}$$

where *C*_e_ (mg L^−1^) is the amount of quercetin in solution and *q*_e_ (mg g^−1^) and *q*_max_ (mg g^−1^) are the equilibrium and maximum adsorption capacities of the adsorbent, respectively. *k*_L_ (L mg^−1^) is the Langmuir constant, which represents the affinity of the binding site and the adsorption energy. Langmuir parameters such as *K*_L_ and *q*_max_ are calculated from the intercept and slope of the linear plot of *C*_e_/*q*_e_ vs. *C*_e_. *K*_F_ (L mg^−1^) and *n* are Freundlich constants representing adsorption capacity and adsorption intensity, respectively, and are calculated from the linear plot of log *q*_e_ versus log *C*_e_.

The Langmuir isotherm assumes that the binding sites on the surface of sorbent are homogeneous, and that adsorption occurs in a monolayer pattern with no interaction between the adsorption sites. In contrast, the Freundlich isotherm assumes that the adsorption process is based on a heterogeneous surface and that adsorption occurs in multiple layers.  Table 1 shows the parameters for the two models. On the bases of fitted curves and data, it can be seen that the *R*^2^ of the linear Langmuir (0.9959), is higher than that of Freundlich (0.9703). Thus, the Langmuir model best describes the adsorption process of quercetin and confirms that the adsorptive sites are homogeneous on Fe_3_O_4_@ GO@mSiO_2_.

### 3.4. Selectivity of Fe_3_O_4_@GO@mSiO_2_ Nanosorbent

The influence of foreign species on the determination of quercetin was studied under optimized conditions. For this purpose, sample solutions containing 500 ng mL^−1^ quercetin and different concentrations of interfering species were prepared. From the experimental results presented in Table 2, it can be seen that when the extraction amount of the analyte is changed by more than 5%, most of the cations can be retained in the pores of the adsorbent and reduce the absorption of quercetin. Organic compounds with relatively similar structures, such as gallic acid and caffeine, can also be adsorbed. It can be concluded that the adsorbent used in this project has relatively good performance for quercetin but is not very selective. To increase the selectivity, the silanol groups in the pores of the adsorbent need to be changed.

### 3.5. Method Validation

Under the optimized conditions, the performance of the developed method for the determination of quercetin was investigated according to the measurement procedure. The calibration curve was linear in the range of 20–800 *μ*g L^−1^. The limit (LOD) was 5.2 *μ*g L^−1^ and was calculated using the equation LOD = 3Sb/m, where Sb and m are the standard deviations of three blank repeats and the slope of the calibration curve, respectively. Additional performance data for this method, including recovery and preconcentration factor, are shown in Table 3. In addition, a comparison of the proposed method with the other reported preconcentration methods for the extraction of quercetin from real samples was made in Table 4. The results show that the proposed method for the determination of quercetin provides superior or comparable adsorption capacity for quercetin compared to the literature reports.

### 3.6. Real Sample Analysis

The method was used to determine quercetin in samples of spinach, green bell pepper, red onion, and dill weed by the standard addition method. Different concentrations of quercetin were added and analyzed by the proposed method. As shown in Table 5, recoveries ranging from 94.00 to 107.00% and RSD values ranging from 2.43 to 4.49 were obtained by the proposed method. These results indicate that the proposed method is suitable for the separation and preconcentration of quercetin from real samples.

## 4. Conclusions

In this study, an attempt was made to prepare an adsorbent of Fe_3_O_4_@GO@mSiO_2_ with graphene oxide properties as a nanosheet and porous mesoporous silica with magnetic properties by the Hammer method to extract quercetin and analyze it by a simple spectroscopic method. The prepared sorbent was characterized by FTIR, FESEM, EDX, XRD, VSM, and XPS techniques. The pH of the aqueous solution as a parameter affecting the interaction between quercetin and the synthesized adsorbent showed that a strongly acidic or alkaline environment was not suitable due to the reduction of electrostatic interaction and hydrogen bonding between the analyte and the sorbent. The results showed that the synthesized magnetic nanoparticle sorbent can adsorb and extract small amounts of quercetin from aqueous solutions under optimal conditions, nonselectively. It was successfully used to measure quercetin in real plant samples (spinach, green pepper, dill, and red onion).

## Figures and Tables

**Table 1:** Parameters of Langmuir and Freundlich equations.

Langmuir			Freundlich		
*K*_*L*_	*q*_max_	*R* ^2^	*K*_*F*_	*n*	*R* ^2^
2.11	1.3	0.9959	0.797	3.19	0.9703

**Table 2:** Analytical characteristics of the proposed method.

Figures of merit	Quercetin
*R* ^2^	0.9964
Linear range (*μ*g L^−1^)	20–800
LOD (*μ*g L^−1^)	5.2
RSD (*n* = 3)	3.78%
Recovery (%)	94%
*F* (*C*_ext_/*C*_0_)	4.71

**Table 3:** Effect of interfering species in the presence of 500 *μ*g L^−1^ quercetin.

Coexisting substance	Tolerance limit
Ni(NO_3_)_2_.6H_2_O	5
Hg(NO_3_)_2_.H_2_O	
Cd(NO_3_)_2_.4H_2_O	

Cr(NO_3_)_3_.9H_2_O	10
Al(NO_3_)_3_.9H_2_O	
Cu(NO_3_)_2_.3H_2_O	
Mn(NO_3_)_2_.4H_2_O	
Gallic acid	

Ca(NO_3_)_2_.4H_2_O	20

La(NO_3_)_3_.6H_2_O	15

Caffeine	25

**Table 4:** Comparison of the proposed method with some different methods to determination of quercetin.

Methods	Sample	LOD (*μ*g L^−1^)	LDR (*μ*g L^−1^)	RSD (%)	Reference
GO/Fe_3_O_4_/HPLC-UV	Tea and urine	0.8 (*μ*g L^−1^)	3–1500 (*μ*g L^−1^)	≤5.6	[18]

d-MSPE^**1**^/Fe_3_O_4_/SiO_2_/HPLC-UV	Fruit juices and vegetables	0.18 (*μ*g L^−1^)	0.6–500 (*μ*g L^−1^)	0.80	[19]

DLLME-SFO^**2**^/UV-Vis	Apple, onion, and tomato	3.02 (*μ*g L^−1^)	15.10–151.00 (*μ*g L^−1^)	2.8	[20]

MIP-SPME^**3**^/HPLC-UV	Black tea Green tea Coffee	9.94 (*μ*g L^−1^)	50–100.000 (*μ*g L^−1^)	2.3	[21]

MSPE (MACC^**4**^)/UV-Vis	Onion	1.40 (*μ*g L)	16.8–3358 (*μ*g L^−1^)	<10	[22]

SPME (CW/TPR^**5**^) HPLC-UV	Food sample	9.94 (*μ*g L^−1^)	50–100 000 (*μ*g L^−1^)	2.3	[23]

MSPE (Fe_3_O_4_@GO@mSiO_2_)/UV-Vis	Spinach Green pepper Red onion Dill weed	5.2 (*μ*g L^−1^)	20–800 (*μ*g L^−1^)	3.78	This work

^**1**^Magnetic solid-phase extraction. ^**2**^Dispersive liquid-liquid microextraction based on the solidification of the coating organic. ^**3**^Molecularly imprinted polymer solid-phase microextraction. ^**4**^Magnetic-activated carbon cloth. ^**5**^Carbowax/templated resin.

**Table 5:** Determination of quercetin in real samples.

Sample	Added (*μ*g g^−1^)	Found (*μ*g g^−1^)	Recovery (%)	RSD (*n* = 3)
Spinach	0	13.61	—	4.01
	5	18.36	95.00	2.43
	10	23.92	103.10	3.98

Green pepper	0	3.87	—	2.98
	1	4.81	94.00	4.49
	3	6.92	101.67	3.76

Red onion	0	5.71	—	4.21
	1	6.78	94.67	3.03
	3	8.55	107.00	3.20

Dill weed	0	9.93	—	2.88
	2	11.98	102.50	2.74
	5	14.65	94.40	3.06

## Data Availability

Our research data during this study are included in the article.

## References

[1] Pinheiro R. G., Pinheiro M., Neves A. R. (2021). Nanotechnology innovations to enhance the therapeutic efficacy of quercetin. *Nanomaterials*.

[2] He Y., Yi C., Zhang X., Zhao W., Yu D. (2021). Magnetic graphene oxide: synthesis approaches, physicochemical characteristics, and biomedical applications. *TrAC, Trends in Analytical Chemistry*.

[3] Maciel E. V. S., Mejía-Carmona K., Jordan-Sinisterra M., Da Silva L. F., Vargas Medina D. A., Lanças F. M. (2020). The current role of graphene-based nanomaterials in the sample preparation arena. *Frontiers in Chemistry*.

[4] Tran C. V., Quang D. V., Nguyen Thi H. P., Truong T. N., La D. D. (2020). Effective removal of Pb (II) from aqueous media by a new design of Cu–Mg binary ferrite. *ACS Omega*.

[5] Huguet-Casquero A., Gainza E., Pedraz J. L. (2021). Towards green nanoscience: from extraction to nanoformulation. *Biotechnology Advances*.

[6] Alboghbeish M., Saghanezhad S. J., Larki A. (2022). Piperazine-modified magnetic graphene oxide (Pip@ MGO) as a novel nanocomposite for the effective removal of lead ions; using RSM optimization. *Scientific Reports*.

[7] Ma Y. X., Shao W. J., Jin P. S., Kou Y. L., Li X. (2019). Magnetic graphene oxide grafted with different hydrophobic chain lengths low‐generation polyamidoamine dendrimers for adsorption of Pb (II) and Hg (II) in aqueous solution. *Polymer Composites*.

[8] Sun L. (2019). Structure and synthesis of graphene oxide. *Chinese Journal of Chemical Engineering*.

[9] Xiao H., Cai L., Chen S., Zhang Z. (2020). Magnetic mesoporous silica/graphene oxide based molecularly imprinted polymers for fast selective separation of bovine hemoglobin. *SN Applied Sciences*.

[10] Mustafa I. (2019). Methylene blue removal from water using H_2_SO_4_ crosslinked magnetic chitosan nanocomposite beads. *Microchemical Journal*.

[11] Pourjavadi A., Tehrani Z. M., Jokar S. (2015). Functionalized mesoporous silica-coated magnetic graphene oxide by polyglycerol-g-polycaprolactone with pH-responsive behavior: designed for targeted and controlled doxorubicin delivery. *Journal of Industrial and Engineering Chemistry*.

[12] Yusuf M., Kumar M., Khan M. A., Sillanpää M., Arafat H. (2019). A review on exfoliation, characterization, environmental and energy applications of graphene and graphene-based composites. *Advances in Colloid and Interface Science*.

[13] Huang T., Yan M., He K. (2019). Efficient removal of methylene blue from aqueous solutions using magnetic graphene oxide modified zeolite. *Journal of Colloid and Interface Science*.

[14] Li Z., Zhang Y., Feng N. (2019). Mesoporous silica nanoparticles: synthesis, classification, drug loading, pharmacokinetics, biocompatibility, and application in drug delivery. *Expert Opinion on Drug Delivery*.

[15] Bourebrab M. A., Oben D. T., Durand G. G., Taylor P. G., Bruce J. I., Bassindale A. R. (2018). Influence of the initial chemical conditions on the rational design of silica particles. *Journal of Sol-Gel Science and Technology*.

[16] Murugan B., Sagadevan S., Lett A. (2020). Role of mesoporous silica nanoparticles for the drug delivery applications. *Materials Research Express*.

[17] Soylak M., Ozdemir B., Yilmaz E. (2020). An environmentally friendly and novel amine-based liquid phase microextraction of quercetin in food samples prior to its determination by UV–vis spectrophotometry. *Spectrochimica Acta Part A: Molecular and Biomolecular Spectroscopy*.

[18] Wu J., Xiao D., Zhao H. (2015). A nanocomposite consisting of graphene oxide and Fe_3_O_4_ magnetic nanoparticles for the extraction of flavonoids from tea, wine and urine samples. *Microchimica Acta*.

[19] Sani T. H., Hadjmohammadi M., Fatemi M. H. (2020). Extraction and determination of flavonoids in fruit juices and vegetables using Fe_3_O_4_/SiO_2_ magnetic nanoparticles modified with mixed hemi/ad‐micelle cetyltrimethylammonium bromide and high performance liquid chromatography. *Journal of Separation Science*.

[20] Asadollahi T., Dadfarnia S., Haji Shabani A. M., Amirkavei M. (2015). Separation/preconcentration and determination of quercetin in food samples by dispersive liquid–liquid microextraction based on solidification of floating organic drop-flow injection spectrophotometry. *Journal of Food Science and Technology*.

[21] Rahimi M., Bahar S., Heydari R., Amininasab S. M. (2019). Determination of quercetin using a molecularly imprinted polymer as solid-phase microextraction sorbent and high-performance liquid chromatography. *Microchemical Journal*.

[22] Arain M. B., Yilmaz E., Hoda N., Kazi T. G., Soylak M. (2019). Magnetic solid-phase extraction of quercetin on magnetic-activated carbon cloth (MACC). *Journal of the Iranian Chemical Society*.

[23] Kumar A., Malik A. K., Tewary D. K. (2009). A new method for determination of myricetin and quercetin using solid phase microextraction–high performance liquid chromatography–ultra violet/visible system in grapes, vegetables and red wine samples. *Analytica Chimica Acta*.

